# A Companion Robot for Children With Asthma: Descriptive Development and Feasibility Pilot Study

**DOI:** 10.2196/68943

**Published:** 2025-06-23

**Authors:** Jinnaphat Sangngam, Somsiri Rungamornrat, Rungnapa Santipipat, Kunchira Ponthanee

**Affiliations:** 1 Pediatric Nursing Department, Faculty of Nursing, Mahidol University, 2 Wang Lang Road, Siriraj, Bangkok Noi, Bangkok, 10700, Thailand, 66 24197469; 2 Faculty of Medicine, Siriraj Hospital, Mahidol University, Bangkok, Thailand

**Keywords:** companion robot, app, asthma, children, caregiver, model

## Abstract

**Background:**

Consistent medication use and proper inhaler technique are essential in pediatric asthma, and young children require supportive tools to maintain these practices.

**Objective:**

This study aimed to investigate the caregivers’ ability to use a companion robot–assisted app for children with asthma, their attitudes toward the usage, and the characteristics that hinder or facilitate the implementation.

**Methods:**

This study employed a descriptive design. The sample group consisted of 30 children with asthma aged 3‐6 years who received treatment at an asthma clinic and their caregivers. The companion robot for children with asthma called “Pukkabot,” which is an innovation that is developed to teach inhalation techniques and to raise awareness about consistent medication administration through positive reinforcement, was examined. Data collection included personal information questionnaires, the System Usability Scale (SUS) for evaluating usage and overall satisfaction, and interviews to gather attitudes toward apps and characteristics that hinder or facilitate. Data were analyzed with descriptive statistics and content analysis.

**Results:**

The study revealed that the scores for usability and overall satisfaction were 80.6, which is above the standard threshold and rated at grade A or an excellent level. Additionally, caregivers stated that the companion robots were easy to use, not complicated, had appropriately sized screens, and demonstrated clear images and sounds. The detailed steps for inhalation were exhibited, and reminders included those of medication times. The children with asthma enjoyed the app and were very interested, making most caregivers want to continue their use.

**Conclusions:**

Caregivers were generally satisfied with the usability, finding it easy to use and engaging, which successfully attracted the interest of the children with asthma. Therefore, the companion robot may be used further, with the following recommendations: improving its physical design, adding content, incorporating tracking and symptom assessment systems, and creating a downloadable mobile app for greater accessibility and convenience.

## Introduction

Asthma is a chronic respiratory condition that is characterized by bronchial tube inflammation, causing a heightened response to allergens and environmental factors. This causes symptoms, including bronchial hyperreactivity, bronchoconstriction, swelling, and increased mucus production [1]. Asthma affects individuals of any age, but it is particularly prevalent in children under 6 years of age, who are more susceptible to respiratory infections that worsen bronchial inflammation. Early detection and effective management of asthma exacerbations are crucial for preventing chronic inflammation and improving long-term health outcomes as children develop. The World Health Organization reported that approximately 262 million people globally experience difficulties from asthma, with a significant proportion being children from low- to middle-income countries, where the prevalence is increasing [2]. Approximately 2 million children with asthma are affected in Thailand, with 9.8% experiencing exacerbations annually and a significant cost associated with treatment [3].

The Global Initiative for Asthma (GINA) provides comprehensive guidelines for pediatric asthma management, focusing on symptom control through medication, decreasing environmental trigger exposure, and continuous symptom monitoring [4]. The key treatment goals include achieving satisfactory symptom control, minimizing acute exacerbations, allowing normal daily activities, and maintaining optimal lung function. Children with asthma require both relief medications for acute symptoms and long-term controller medications to prevent future exacerbations and maintain symptom control [4]. However, many children face difficulties with consistent and correct inhaler use, which is crucial for effective medication delivery [5,6]. Literature emphasizes issues, such as incorrect inhaler techniques and irregular medication adherence, frequently stemming from caregivers’s misconceptions about inhalers, fears of side effects, and concerns over costs [6,7]. Therefore, both children and their caregivers need to receive a thorough education on proper inhaler use and the significance of consistent medication adherence.

Environmental pollutants, particularly particulate matter (PM) with a diameter of <2.5 microns (PM 2.5), are significant triggers for asthma exacerbations, causing bronchial inflammation and irritation [8]. Children with asthma who are exposed to high air pollution levels may experience worsened symptoms, which impair lung function and require emergency medical interventions [9,10]. Various smartphone apps provide real-time air quality data, but challenges associated with accessibility and technological literacy among caregivers may limit their effectiveness in managing asthma triggers [11]. Effective asthma management requires collaboration between the pediatric patient and their caregiver, tailored to the child’s developmental stage. Instilling health-promoting behaviors during this critical period facilitates the teaching of consistent inhaler use for preschool-aged children.

Recent technological advancements have resulted in the creation of devices aimed at improving inhaler use among children with asthma [12]. In particular, the JOE robot, which assists with demonstrating inhaler techniques, currently supports only English and French, limiting its accessibility for users who speak other languages [13]. Other innovations, such as the Whizz smart inhaler [14] and CapMeDicTM [15], provide various features to encourage proper inhaler utilization but may not fully engage young children or be widely available in Thailand. Hence, Thammasat University (Thailand) has developed the “Asthma Care” app, which provides comprehensive guidance on asthma management. However, further innovations that are specifically designed for preschool-aged children to meet their unique needs are highly warranted.

This study aimed to assess caregivers’ ability to use “Pukkabot,” a companion robot-assisted app designed to teach correct inhaler techniques and promote consistent medication adherence through positive reinforcement, considering the necessity for accessible and effective asthma management tools for young children. Additionally, the study investigated caregivers’ attitudes toward “Pukkabot,” determining features that may either facilitate or hinder its use. “Pukkabot” seeks to improve symptom control, reduce severe asthma attacks, and enhance overall treatment efficacy by fostering early awareness and proper inhaler techniques in children with asthma, ultimately minimizing hospitalizations and lowering the risk of severe complications. The results from this research provided valuable information into the development of effective and user-friendly asthma management tools for young children in Thailand, contributing to improved health outcomes and quality of life for children with asthma.

## Methods

This descriptive study aimed to explore the feasibility of using “Pukkabot,” and to explore barriers and facilitators to its use.

### Recruitment Process

This study recruited caregivers and children with asthma from the Allergy and Immunology Clinic in the asthma clinic, Pediatric Outpatient Department of a tertiary hospital from February to September 2024. The children included were aged 3‐6 years, physician-diagnosed with asthma of any severity for at least 6 months, receiving treatment with at least 1 inhaled medication, and without a history of uncertain clinical symptoms or learning disabilities. Caregivers were required to be ≥18 years old and related to the child.

A convenience sample of 30 children with asthma was recruited for this single-arm interventional trial. The sample size was guided by recommendations for pilot and feasibility studies [16]. According to Brooke [17], a general rule of thumb is to include at least 30 participants to allow for reasonable estimation of study parameters.

### Study Design

The researcher instructed the caregiver and the child to interact with the companion robot. The process began with the caregiver setting a medication reminder (set for 5 min) and selecting an air quality monitoring option within the app, which displays the real-time Air Quality Index (AQI) to raise awareness about environmental triggers. Afterward, the child was invited to select a preferred pet (rabbit, dog, cat, bear, and dragon). When the reminder alarm was triggered, the child practiced medication administration steps by following on-screen instructions, using an empty inhaler device that contained no active medication. This activity was conducted solely as a demonstration to simulate proper inhaler technique, without actual medication use. The demonstration lasted approximately 5 minutes, during which the researcher provided verbal guidance and support. The entire process was completed within approximately 15 minutes.

Afterward, the caregivers were instructed to complete a 10-item System Usability Scale (SUS) regarding the companion robot. The caregivers completed the questionnaire, whereas the researcher recorded personal information from the patient’s medical records.

Finally, the researcher interviewed the caregivers to gather their attitudes toward using the asthma management companion robot, as well as any perceived barriers or facilitators to its implementation. This aimed to determine areas for system improvement. The interview included 6 questions and lasted approximately 5‐10 minutes.

### Research Instruments

In this study, various instruments were used to gather data effectively, ensuring a comprehensive understanding of the participants’ backgrounds and experiences. The instruments included personal information questionnaires, patient medical record data forms, the SUS, and caregiver interviews focused on attitudes toward asthma management companion robot-assisted app for children with asthma.

#### Personal Information Questionnaires

The interview form designed for children with asthma collected vital information, including age, sex, age at diagnosis, prescribed medications, comorbid conditions, and asthma exacerbation-related hospitalization history. This comprehensive data helped develop a baseline profile of the pediatric participants, enabling a nuanced analysis of their health status and treatment history.

The personal information interview form for caregivers collected critical details, including age, sex, educational level, marital status, occupation, income, and relationship to the pediatric patient. Understanding the caregivers’ backgrounds is crucial, as their socioeconomic status, educational level, and relationship to the child significantly affect asthma management strategies and the usage of health care resources.

#### Patient Medical Record Data Form

The Patient Medical Record Data Form was used to collect clinical data directly from the medical records of children with asthma. This form provided comprehensive information regarding asthma severity, which was classified according to the GINA guidelines by health care professionals, age at diagnosis, prescribed medications for symptom control and relief, other treatments received, comorbid conditions, and any asthma-related hospitalization history.

#### System Usability Scale (SUS)

The SUS, developed by John Brooke in 1986 [17], is a widely recognized tool for evaluating the usability and overall satisfaction of a companion robot-assisted app. The SUS consists of a 5-point Likert scale, with response options ranging from 1 (“strongly disagree”) to 5 (“strongly agree”). The scale includes 10 items—5 positively worded and 5 negatively worded—that assess the usability of the asthma management app. Scoring involves assigning values from 1 to 5 for the positive items while applying reverse scoring for negative items. The total score is calculated and then multiplied by 2.5 to convert the range from 0 to 40 to a scale of 0‐100. A score of ≥68 indicates acceptable usability, corresponding to the 50th percentile, whereas scores below this threshold indicate areas for improvement in design and usability [17-19].

#### Caregiver Interviews on Attitudes Toward Using a Companion Robot for Asthma Management

In-depth interviews were conducted with a semistructured format to gain information about caregivers’ perceptions of the asthma management app and the pediatric asthma model. This approach facilitated a nuanced exploration of caregivers’ attitudes, including barriers and facilitators to app. The interview consisted of 7 main questions developed to prompt discussion on previous experiences with similar technologies, willingness to use the asthma model, and opinions on the model’s design. In particular, caregivers were asked the following questions: “Have you ever encountered a similar app or model before?”; “If not, after learning about or using this system, would you consider using this asthma model for your child?”; “What are the main reasons for your decision?” Another sample question included the following: “Which aspects of the model’s design do you think are not well-developed or convenient for use?” The interviews were used to collect qualitative data that complemented the quantitative results, providing information on how to improve the effectiveness and user acceptance of asthma management tools.

In summary, the diverse range of research instruments used in this study—personal information questionnaires, patient medical record data forms, the SUS, and caregiver interviews—enabled a comprehensive assessment of both the clinical and experiential aspects of asthma management in children. The research aimed to provide actionable information for improving asthma care delivery and technology usage among caregivers and health care professionals by integrating quantitative and qualitative data.

### Pukkabot

**Figure 1.**
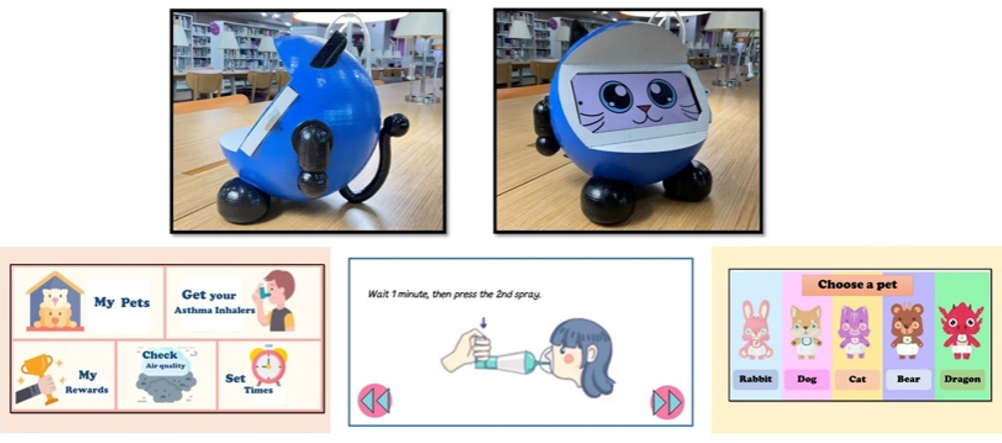
Appearance of the Pukkabot companion robot and screenshots of its mobile app interface designed to support caregivers of children with asthma.

The tool used in this study is the companion robot-assisted app for children with asthma, named “Pukkabot,” an innovative device designed to teach proper inhaler use and promote medication adherence through positive reinforcement (Figure 1). The design of the companion robot is tailored to each child’s treatment plan, specifically focusing on those who require both inhaled controller medications and quick-relief medications. The app operates on the Android 13 platform, featuring a touch-sensitive screen that displays both video and audio content. The model, shaped like an animal, measures 23.5 cm in width, 23.1 cm in length, 21 cm in height, and 800 g in weight, including the screen. Its structure is composed of polylactic acid plastic, providing strength and lightweight durability during use. Polylactic acid is a form of plastic derived from natural sources, making it non-irritating during use. It can be cleaned with an alcohol spray. The model is developed to be placed on a flat surface, with the weight supported by its 2 legs and tail for balance while using the screen. The screen area is recessed by approximately 2 inches to prevent damage in case of impact or if it falls from a height. The main display screen provides the following 5 operational modes.

1. My pet: the user must select a pet by tapping on the desired pet when entering for the first time. Afterward, the screen will display the virtual pet. This screen will enable the user to feed the pet, which receives 1 food item per completed inhaler use.
2. Timer: the screen displays a timer to remind the user of the medication inhalation schedule. The screen shows an image of the eyes when in standby mode. The screen changes to display the message “It’s time to recharge” along with a cartoon sound alert as the scheduled inhalation time approaches. The timer can be set for 2 reminders per day.
3. Inhaler use: this displays images and step-by-step instructions for using the inhaler, with accompanying audio. Users navigate through the steps via control buttons, enabling them to administer medication in real time alongside the instructions displayed.
4. Rewards: this displays the rewards earned, with the virtual pet growing according to the number of completed inhaler uses and the food it receives. This is designed to motivate children to use their inhalers consistently.
5. Air quality: this displays the local air quality in terms of AQI levels. It is used along with the daily usage of inhalers to alert the user of the severity and provide information on avoiding triggers.

In total, 1 pediatric nurse with expertise in asthma care and 1 pharmacist with expertise in respiratory care validated the “Pukkabot” for content accuracy, structure, and language. Feedback from these experts was incorporated to refine and improve the system.

### Ethical Considerations

The study was conducted in accordance with the Declaration of Helsinki and was approved by the Institutional Review Board Committee of the Faculty of Nursing and the Faculty of Medicine, Siriraj Hospital, Mahidol University (MU-MOU CoA no. IRB-NS2023/824.1212, dated December 12, 2023). All caregivers and children were informed of the study’s purpose and procedures and provided written informed consent before participation. Data were anonymized prior to analysis to protect the confidentiality and privacy of participants. No financial compensation was provided; however, caregivers received small tokens of appreciation in recognition of their time and participation.

### Data Analysis

Personal data and usability assessment results were analyzed with descriptive statistics. Content analysis was conducted to assess the system’s usability, determining both barriers and facilitators based on caregiver interviews.

## Results

### General Characteristics of Children With Asthma and Their Caregivers

Among the children with asthma, 63% (19/30) were male and 37% (11/30) were female, with an average age of 5.3 (SD 0.7) years. The onset of asthma occurred at an average age of 2.9 (SD 1.2) years, with 77% (23/30) of the children being diagnosed with allergic rhinitis. A majority (21/30, 70%) of children used both the controller and reliever inhaler medications. Only 7% (2/30) of the children had partly controlled asthma, whereas the majority (28/30, 93%) demonstrated well-controlled asthma. Asthma control was classified according to GINA guidelines, with “well-controlled” defined as symptoms occurring no more than twice per week, minimal nighttime symptoms, infrequent use of rescue medication, and no limitations on daily activities.

Of the caregivers of children with asthma, 80% (24/30) were female, with an average age of 40.7 (SD 6.8) years. A large proportion (22/30, 73%) were mothers, with only 7% (2/30) being grandmothers. Regarding educational background, 50% (15/30) completed a bachelor’s degree, whereas 20% (6/30) completed a master’s degree. Regarding occupations, 57% (17/30) were employed. Most caregivers (19/30, 63%) reported a monthly household income of >฿20,000 (approximately US $570), which is considered a moderate-income level and is above the country’s median household income.

### Usability and Overall Satisfaction

The average SUS score from the 30 caregivers was 80.6 (SD 12.2), with scores of 52.5‐97.5 based on the results of the usability and satisfaction assessment, as measured by the SUS. These scores are higher than the standard benchmark of 68, indicating a level of usability above average. The SUS score is categorized in the grade A range, indicating a high level of usability and acceptability.

The highest rated aspects of usability and overall satisfaction in reviewing the individual items were item 7, “I believe others will quickly understand how to use this app,” and item 3, “I find this app easy to use.” In contrast, the lowest rated aspect was item 4, “I believe I need assistance to use this app,” indicating that most users did not feel the need for additional help and could use the app independently.

### Attitudes Toward Using a Companion Robot for Asthma Management

The results in this section depend on interviews about attitudes toward the use of the companion robot-assisted app for children with asthma, as well as user behavior observations. The results are categorized into 3 main areas: attitudes toward use, barriers and facilitators, and improvement suggestions.

#### Attitudes Toward the Use of the Companion Robot

Caregivers’ experiences and perspectives on the tools were largely positive.

There was a lack of previous experience. None of the caregivers had previous experience with similar apps for asthma management, although 3 had learned about inhaler use through videos or YouTube.

There were also positive reactions to the companion robot. After being introduced to the tools, 26 (87%) caregivers expressed an interest in using them. They considered the tools easy to use and believed they could motivate their children to use their inhalers regularly. The cute, pet-themed design was particularly appealing to the children, making the process more engaging.

I’ve never used it before, but I would like to. My child and I have used the inhaler for a long time, but this doll could motivate my child to want to use it more. I like that it has a pet, which makes it fun for my child and serves as good motivation.

[Mother, aged 38 years]

*I haven’t used it, but I’ve watched videos on how to utilize the inhaler and have an app that monitors air quality, although I rarely check it. This seems useful, and my child seems interested. It may help motivate my child to use the inhaler properly, as they frequently avoid it. I like the presence of a cartoon pet to capture their attention.*

[Mother, aged 30 years]

#### Barriers and Facilitators in the Use of Companion Robot

Caregivers identified both positive aspects and challenges to the use of the companion robot. The feedback is categorized into 2 sub-themes: (1) external features of the companion robot and (2) usability.

##### External Features of the Companion Robot

In terms of facilitators, 9 (30%) caregivers felt that the model’s design was appealing and stable, with a pet-like appearance that caught children’s interest.

In terms of barriers, 21 (70%) caregivers emphasized several design issues, including that the model was too large to be portable, its surface was not smooth, and the colors were not vibrant.

*It demonstrates a complete range of functions, but the size is a drawback. It is too large to carry around, and an app would be more convenient. I think the app would only be initially engaging for children unless additional features are added.*

[Mother, aged 43 years]

##### Usability

In terms of facilitators, all caregivers reported that the app was easy to use. The content, images, and audio instructions were concise and suitable for young children, enabling them to follow the steps independently. The cute cartoons and pet-care game kept the children motivated to regularly use their inhalers. Additionally, the app included helpful features, such as medication reminders, air quality monitoring, and health guidelines. Most (26/30, 87%) caregivers felt that the light, colors, and sounds of the app exhibited no negative effect on children’s eyesight due to the short usage duration.

In terms of barriers, some (3/30, 10%) caregivers stated that the medication preparation steps were too long and that users had to manually advance through the inhaler steps. In total, 4 caregivers (4/30, 13%) expressed concerns about screen addiction from using the app frequently.

*I like the reminder feature for medication timing, as it helps us not forget. The model’s large size is inconvenient to move, and I would prefer it as a mobile app. I’d also like a feature to count the number of doses administered.*

[Mother, aged 38 years]

*I like the cartoon that captures my child’s attention, and the images and sounds are clear. I appreciate the advice on when to use emergency inhalers according to breathing difficulty. A mobile app would be more convenient.*

[Father, aged 37 years]

### Suggestions for Improving the Companion Robot

Caregivers provided recommendations to improve both the companion robot for asthma management:

- App development: a majority (27/30, 90%) of caregivers recommended developing the app into a smartphone-compatible version for easier access.
- Model design: a smaller group (3/30, 10%) indicated retaining the model but making it more portable by adjusting the design to be rectangular and flat for easier storage.
- Functional enhancements: several additional features were proposed, including (1) instructions for cleaning the inhaler device, (2) tracking inhaler use and remaining medication doses, (3) an action plan for asthma exacerbations, (4) videos showing asthma symptoms, (5) videos demonstrating nasal irrigation techniques, (6) notifications about environmental triggers (eg, high dust levels), and (7) contact channels for health care teams.

## Discussion

### Principal Findings

The study revealed that caregivers reported an average post-use satisfaction score of 80.6 (SD 12.2) on the SUS, which exceeds the standard threshold and falls within the “Grade A” category. This high score indicates that caregivers of children with asthma were highly satisfied after their initial trial of the companion robot. Such a positive reception may affect their decision to continue using these tools in the future. Further analysis revealed that most caregivers expressed satisfaction due to the app’s ease of use, its simplicity, and because they did not require assistance during the operation.

Interviews with caregivers indicated that none had previous experience using an app or model specifically designed for children with asthma, although some had learned inhalation techniques from videos or web-based media primarily intended for adults. However, all caregivers considered the app user-friendly, with a comprehensive set of functions. The content, audio, and animated illustrations of inhaler usage were deemed suitable for young children, enabling them to independently follow the steps. Additionally, the pet-care game included in the app helped motivate children to consistently use their inhalers. The app can reduce disease severity and improve treatment outcomes, enabling children to live healthy, normal lives, by encouraging early asthma control and fostering self-discipline in inhaler use. Motivation in children under 6 years of age is significantly improved through interactive and engaging tools. Research reveals that interactive learning media, such as game-based learning and multimedia presentations, foster intrinsic motivation by providing dynamic learning experiences that cater to diverse learning styles [20].

A majority of caregivers indicated that the app could be optimized for use on mobile platforms, but the design of the companion robot for children with asthma, “Pukkabot,” in this study specifically aimed to provide preschool-aged children (ages 3‐6 y) with a tangible companion during inhalation therapy. The model functions as a motivator for consistent inhaler use to help control asthma symptoms. Familiar toy-like designs in therapeutic devices improve children’s emotional connections, thereby increasing treatment engagement and adherence, as evidenced by positive feedback from children and parents regarding the respiratory aid for inhalers (RAFIhaler) experience [21].

Caregivers stated that the asthma inhaler details provided in “Pukkabot” aligned well with their requirement for managing children with asthma. This result aligns with research by Lio et al [22], who developed an app for children with asthma aged 2‐12 years. Their study revealed that children and caregivers were more likely to use the app when it was engaging, easy to use, and contained essential content, including disease pathophysiology, self-care practices, and asthma management strategies [22]. Similarly, Li et al [23] established an app for asthma self-management in children that featured symptom evaluation, treatment planning, inhaler usage, and communication with doctors in emergencies, further improving asthma management. Together, these studies emphasize the importance of user-friendly, comprehensive apps in enhancing asthma care for children and their caregivers.

The ability of apps to set multiple reminders for inhaler use significantly benefits children with asthma, particularly those requiring frequent medication doses. A study revealed that setting multiple alarms ensures timely medication adherence, which is crucial for asthma management in children who may require varying inhaler schedules based on the severity of their condition [24]. Additionally, the inclusion of the AQI monitoring function enables both caregivers and children to be vigilant about daily air quality changes. PM 2.5 significantly affects respiratory health, especially in children with asthma [9]. Children with asthma are particularly vulnerable to air pollution such as ozone and PM 2.5, which have been associated with increased respiratory symptoms, hospitalizations, and long-term impairment in lung function [25]. Therefore, it is essential for children and their caregivers to stay informed and take precautions based on daily air quality. This feature enables children and caregivers to make informed decisions about daily activities, including staying indoors, wearing masks to prevent exposure, or adjusting outdoor routines.

In this study, most (22/30, 73%) caregivers were mothers, and 50% (15/30) held a bachelor’s or master’s degree. Furthermore, 20% (6/30) of them were employed, and 57% (17/30) reported a household income higher than the national minimum wage, with 63% (19/30) exhibiting a family income above the country’s median. A study of parental attitudes towards artificial intelligence in children with asthma found that socioeconomic factors influenced technology adoption, with parents from disadvantaged backgrounds more likely to be reluctant to adopt technology [26]. Therefore, socioeconomic factors may affect caregivers’ readiness to adopt technology for health management. Moreover, this research was conducted at a tertiary hospital with access to high-standard care and advanced medical technology. Most caregivers had received ongoing training and education on asthma management for children, which may have contributed to their interest in using technology, such as this app, for at-home care.

Raising awareness and educating caregivers on asthma management in children remains crucial, and health care professionals must prioritize this. Based on the study’s recommendations, additional features, such as instructions for cleaning inhalers, tracking medication usage and remaining doses, establishing action plans for asthma exacerbations, videos demonstrating key symptoms and nasal irrigation techniques, and alerts for environmental triggers, such as air pollution levels, along with direct communication with health care teams, can improve asthma care tools. These improvements will increase the treatment efficacy.

This study highlights the importance of health care professionals in prioritizing caregiver education, emphasizing that improved asthma care tools—with features such as inhaler cleaning instructions, medication tracking, action plans for exacerbations, videos demonstrating key symptoms, and alerts for environmental triggers—can significantly enhance asthma management. These improvements will boost treatment adherence and overall effectiveness, causing better health outcomes for children with asthma, although continuous monitoring remains necessary as they grow older.

### Limitations

One limitation of this study is that it primarily focused on establishing tools for asthma management without fully addressing the individual variability in caregiver readiness and technological literacy. Additionally, the generalizability of the results may be limited due to the specific demographic and technological context of the study participants.

### Suggestions for Future Study

Special attention should be given to the design of these tools for future research, ensuring that they are engaging for children, portable, and nontriggering to prevent exacerbating asthma symptoms. Studies should focus on the long-term effectiveness of these tools and the way they can be seamlessly integrated into routine clinical practice. Additionally, further investigation into the effect of technological innovations on caregiver engagement, particularly across diverse populations and health care settings, is crucial. These tools may improve asthma management, but recognizing that children with asthma will still require continuous monitoring and treatment as they grow older remains important. Therefore, future studies should prioritize establishing adaptable and dynamic educational content that supports asthma care for children across all stages of development.

## Abbreviations

AQI: Air Quality Index
GINA: Global Initiative for Asthma
PM: particulate matter
SUS: System Usability Scale
